# Small Bowel Intussusception Secondary to Metastatic Renal Cell Carcinoma: A Case Report

**DOI:** 10.7759/cureus.44431

**Published:** 2023-08-31

**Authors:** Pedro Barros, Bárbara Oliveira, Rute Pereira, Marco Dores, Aníbal Coutinho

**Affiliations:** 1 Urology, Centro Hospitalar Universitário do Algarve, Faro, PRT; 2 Urology, Centro Hospitalar Universitário do Algarve – Hospital de Faro, Faro, PRT; 3 General Surgery, Centro Hospitalar Universitário do Algarve – Hospital de Faro, Faro, PRT

**Keywords:** intestinal intussusception, surgery, gastro-intestinal metastasis, renal cell carcinoma, acute bowel obstruction

## Abstract

Bowel intussusception is rare in adults and is usually driven by an underlying pathological process affecting the bowel. Renal cell carcinoma (RCC) is the most common type of kidney cancer and its disease course, depending on the initial histology and disease stage, can metastasize to adrenal glands, lungs, bones, brains and contralateral kidney that can be challenging to follow.

We present the case of a patient with a history of radical left nephrectomy for RCC that developed an acute bowel obstruction, secondary to an ileal metastasis of RCC. In previous surgeries, small bowel obstruction (SBO) is usually found due to adhesions, nonetheless in a patient with a history of high-grade disease at diagnosis, one must keep in mind the possibility of disease relapse in the setting of SBO.

## Introduction

Bowel intussusception refers to the telescoping of a proximal segment of the gastrointestinal tract (GI tract) within the lumen of the adjacent segment. It is more frequent in children, whose treatment is conservative many times.

In adults, who have a lower incidence rate (5% of all intussusceptions and only 1% of all SBO), usually, there's an underlying lesion explaining the intussusception (benign tumors, malignant tumors and idiopathic etiologies as 37.4%, 32.9% and 15.1%, respectively). Given the non-specificity of the symptoms, sometimes it bears a late diagnosis and surgical intervention is usually advised [1-3].

Renal cell carcinoma (RCC) accounts for 3% of all adult cancers, urologic cancer being the most lethal. Given today’s early detection on routine exams, the triad of hematuria, flank pain and fever is rare. Of the newly diagnosed RCC with clinically localized disease, 20-40% of patients will develop metastasis. Metastases originating from RCC infrequently occur in the small intestine and typically manifest as either obstructive symptoms or iron deficiency anemia caused by covert bleeding. Metastatic RCC causing intussusception of the small bowel is an uncommon occurrence with limited documentation in the medical literature [1,2,4].

We present a case study of a patient who was diagnosed with metastatic renal carcinoma in the small bowel and presented to the Emergency Department exhibiting obstructive symptoms caused by intussusception.

## Case presentation

Here we present a case of a female patient, 51 years old, with a history of open left nephrectomy for RCC. The histology showed a clear cell carcinoma with invasion of the renal vein (16.5 cm biggest axis, pT3b, ISUP 4). She had disease progression at one year of follow-up with pulmonary metastasis and paramediastinal mass, already having been submitted to anti-algic radiotherapy and on palliative Sunitinib.

One year and four months after the initial surgery, she presented to the Emergency Department (ER) with a history of colic abdominal pain for the last two days, progressively worsening, and becoming constant and localizing to the right inferior quadrant of the abdomen. She also described anorexia for the last three days and vomits for the last two days. She also reported stopping to pass fecal content the day before in the morning. Her vital signs were stable. Apart from the pain and distended abdomen, the patient revealed no signs of sepsis. The blood gas analysis performed on admission was negative for hyperlactacidemia.

Physical examination revealed a distended abdomen, painful at deep palpation, with signs of peritoneal irritation on the right iliac fossa. The blood analysis revealed a slight anemia (Hgb 10 g/dl), with a normal renal function (Creatinine 0.8 mg/dl).

**Figure 1:**
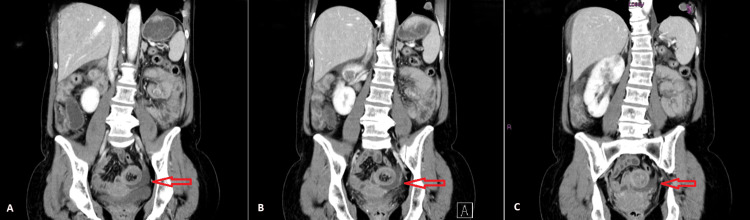
CT scan showing the probable location of the ileo-ileal intussusception with associated free fluid (A-C) and bowel wall thickening (B), and CT findings of intestinal distress. A small residual mass consistent with previous metastasis can also be noted on the inferior lobe of the left lung.

Given the oncologic context and the findings at physical examination, the patient did a computerized tomography (CT) scan that revealed an occlusive process of the ileum by a polypoid neoformation with intestinal intussusception (Figure 1).

**Figure 2:**
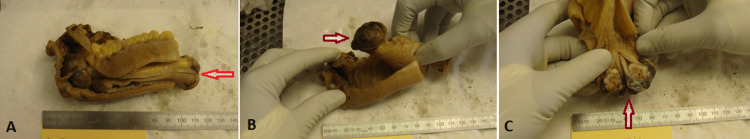
Enterectomy segment showing the ileo-ileal intussusception (A), identifying in the distal segment of the invaginated bowel a tumoral polypoid lesion with 2 cm largest axis (B), and eroded, heterogeneous and brownish mass when cut (C).

The patient was proposed for an emergency exploratory laparotomy that revealed an abnormal mass in the ileum causing the intussusception, and was submitted to a partial enterectomy encompassing the area with primary anastomosis (Figure 2).

She was left on a zero diet on the first 24 hours of post-op period, after which she was allowed to resume liquid intake, with abdominal palpation revealing no signs of peritoneal irritation. Five days later and already on a general diet with normal bowel function, the patient was discharged.

**Figure 3:**
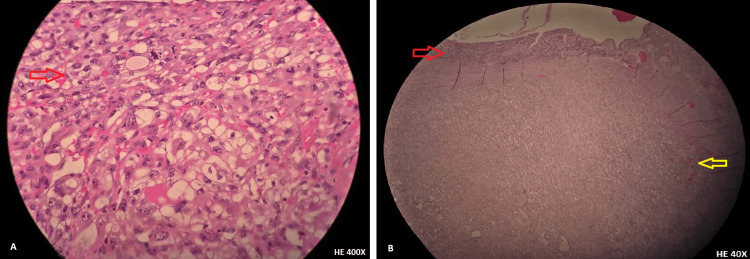
Microscopic examination of the nephrectomy specimen with a clear cell RCC, on the left (A), and the bowel infiltration by a tumoral lesion, consistent with metastasis from RCC clear cell, on the right (B - red arrow showing normal intestinal mucosa; yellow arrow - infiltrating clear cell RCC). RCC: Renal cell carcinoma

The histological analysis of the ileal segment revealed a metastasis from RCC, consistent with the progression of the disease (Figure 3). No immunohistochemical analysis was made. The pathologist preferred the comparison between the first histological product and the enterectomy piece.

Today, at eight months follow-up from the last surgery, the patient has shown pulmonary disease progression and is waiting for approval for Axitinib.

## Discussion

Bowel intussusception is rare in adults (5% of all intussusceptions), and is mainly diagnosed in children, in whom conservative management warrants good results. On the other hand, when it presents in adults, it almost always implies surgical management in order to resolve the obstruction and to find out the mechanism behind it. Even though benign lesions are more frequent as the underlying cause, secondary lesions are also a possibility [1-3].

CT scan is the exam of choice, given its diagnostic accuracy and sensitivity that could reach 80-90% and also the information we can take from it, mainly length, localization, vascular impairment, staging and the existence of a lead point starting the obstruction [1].

The confirmation of the diagnosis is usually made on the operating table. When treating such a condition, many times there is a need for gastro-intestinal resection in order to re-establish normal transit [1].

RCC metastasization can appear long after the surgery and common metastasization sites include the lungs, liver, bones and brain. Viadana et al. in 1976 suggested that metastasization of RCC firstly involves the lung through the renal vein and inferior vena cava. From there, it may spread to anywhere in the body [5]. Only 0.2-0.7% of RCC metastasize to the GI tract (liver excluded) [1,3].

This case is worth noting given the rarity of such presentation of metastatic RCC that could be missed by the practicing physician if one does not keep a high level of suspicion. Diagnosis of GI metastatic lesions from RCC is often delayed - they are discovered as the result of its clinical presentation [1,3]. In a patient with a history of high-grade RCC at diagnosis, though the metastasization being rare to the GI tract, one must keep this in mind in the setting of acute bowel obstruction.

Other cases presenting in the same manner have been treated consistently with enterectomy encompassing the lesion [1]. The prognosis of metastatic RCC is largely dependent on the metastatic route and the response to immunotherapy [4]. This case presents a high-risk patient, whose disease course predisposes her to a bad prognosis from the beginning. The evolution of metastatic pulmonary disease is consistent with the common metastatic route of the kidney [5], but the involvement of the GI tract shows a disease whose control is failing to respond to therapy.

## Conclusions

Metastatic RCC lesions to the GI tract are rare and poorly impact the long-term prognosis. Whenever there is a clinical suspicion of intestinal intussusception in an adult, prompt surgical exploration is advised in order to resolve the obstruction and the underlying defect causing it. This case presents an uncommon metastasization site of RCC, with symptomatic acute intestinal obstruction. It enlightens us to thoroughly revise RCC patients whose disease characteristics prompt them to a higher risk of metastatic progression, keeping in mind the possibility of GI symptoms precluding signs of relapsing.
